# Local microstructure evolution at shear bands in metallic glasses with nanoscale phase separation

**DOI:** 10.1038/srep25832

**Published:** 2016-05-16

**Authors:** Jie He, Ivan Kaban, Norbert Mattern, Kaikai Song, Baoan Sun, Jiuzhou Zhao, Do Hyang Kim, Jürgen Eckert, A. Lindsay Greer

**Affiliations:** 1 Institute of Metal Research, Chinese Academy of Sciences, Shenyang 110016, China; 2 IFW Dresden, Institute for Complex Materials, PO Box 270116, Dresden 01171, Germany; 3 TU Dresden, Institute of Materials Science, Dresden 01062, Germany; 4 Department of Metallurgical Engineering, Center for Noncrystalline Materials, Yonsei University, Seoul 120–749, Korea; 5 Erich Schmid Institute of Materials Science, Austrian Academy of Sciences, Jahnstraβe 12, A-8700 Leoben, Austria; 6 Department Materials Physics, Montanuniversität Leoben, Jahnstraβe 12, A-8700 Leoben, Austria; 7 Department of Materials Science & Metallurgy, University of Cambridge, Cambridge CB3 0FS, UK; 8 WPI Advanced Institute for Materials Research (WPI-AIMR), Tohoku University, Sendai 980-8577, Japan

## Abstract

At room temperature, plastic flow of metallic glasses (MGs) is sharply localized in shear bands, which are a key feature of the plastic deformation in MGs. Despite their clear importance and decades of study, the conditions for formation of shear bands, their structural evolution and multiplication mechanism are still under debate. In this work, we investigate the local conditions at shear bands in new phase-separated bulk MGs containing glassy nanospheres and exhibiting exceptional plasticity under compression. It is found that the glassy nanospheres within the shear band dissolve through mechanical mixing driven by the sharp strain localization there, while those nearby in the matrix coarsen by Ostwald ripening due to the increased atomic mobility. The experimental evidence demonstrates that there exists an affected zone around the shear band. This zone may arise from low-strain plastic deformation in the matrix between the bands. These results suggest that measured property changes originate not only from the shear bands themselves, but also from the affected zones in the adjacent matrix. This work sheds light on direct visualization of deformation-related effects, in particular increased atomic mobility, in the region around shear bands.

Metallic glasses (MGs) have attracted a significant amount of attention due to the fact that they possess unique properties such as high strength, high elastic limit, high wear and corrosion resistance, and excellent soft magnetic behaviour^1^,^2^,^3^,^4^,^5^. However, the limited ductility and especially the immediate catastrophic failure during deformation are major obstacles to applications as structural materials. At room temperature, plastic flow of MGs is sharply localized in small volume regions or so-called shear bands^4^,^5^,^6^,^7^,^8^. These are the carriers of shear in MGs; thus, the material behaviour inside the bands is often treated as key to understanding the mechanism of plastic deformation. This type of deformation and properties of shear bands have been extensively studied using both physical experiments and computer simulations^8^,^9^,^10^,^11^,^12^,^13^,^14^,^15^,^16^,^17^,^18^,^19^,^20^,^21^,^22^. Some understanding of conditions during flow was obtained from sensitive property and structural measurements^10^,^15^,^16^,^21^, high-speed imaging^13^,^14^ and from local temperature rises^22^. Transmission electron microscopy (TEM) and molecular-dynamics (MD) simulations showed that the thickness of shear bands is 10–20 nm^17^,^18^,^19^,^20^. Measurements on the tracer diffusion coefficient in shear bands of a bulk metallic glass revealed an increase of the diffusion rate by more than 8 orders of magnitude with respect to that in the undeformed matrix^15^. Such a drastic acceleration of diffusion has been explained by accumulation of the excess volume inside shear bands observed in theoretical and experimental studies^10^,^15^,^16^,^23^,^24^,^25^. Whereas the properties within shear bands have been qualitatively and quantitatively well studied, the effects on the adjacent matrix are poorly understood.

Cold-worked MGs show increases in enthalpy and volume^23^,^24^,^25^. If these overall property changes were assumed to arise only from changes in the shear bands with 10–20 nm thickness, then the implied changes in the bands could be unreasonably high. Work on a single shear band allows better quantification: an 80-μm-thick slab^10^, cut to include the shear band in the central plane, shows an energy of cold work of 1.7 kJ mol^–1^; if all of this were attributed to a 20-nm-thick band, the stored energy in the shear band would be ~6.8 MJ mol^–1^, nearly 10^3^ times a typical heat of fusion. A similarly unphysical conclusion is reached when considering volume changes. It is inescapable that the volume fraction of material affected by the deformation must exceed that implied by the shear-band thickness seen in TEM. The profiles of reduced hardness suggest a shear-band thickness that is roughly equal to the shear offset on the band^10^. This makes it easier to interpret the enthalpy and volume increases resulting from deformation, but how this effective thickness relates to the flow pattern in shear-band operation is still has remained obscure. Obviously one has to take into consideration also the effects in the matrix nearby the shear bands, which could extend on a micrometre scale as was shown by the measurements of the hardness profile^10^ and the residual strain distribution across shear bands^26^.

In the present work, we report the synthesis of new phase-separated bulk MGs containing glassy nanospheres that are on a scale fine enough to act as a local internal recorder of the flow pattern near a shear band. We provide direct visual evidence for deformation-related effects, in particular increased atomic mobility, in the region around shear bands. Phase separation in MGs can increase compressive plasticity^27^,^28^,^29^,^30^,^31^,^32^,^33^,^34^, raising a concern that the proposed ‘internal recorder’ could alter the deformation mode it is intended to reveal. The observed increased plasticity is, however, not associated with a change in deformation mode, which remains based on distinct shear bands but with an increased population. The length-scale of heterogeneity may be important: a study of partially crystallized MGs found that crystallites with diameters 50–100 nm disrupted shear-banding, but that the banding remained quite distinct for crystallites with diameter ≤20 nm^35^. In the present case, the nanospheres are smaller, with diameters less than the shear-band thickness, and furthermore are glassy not crystalline.

## Results

**Figure 1:**
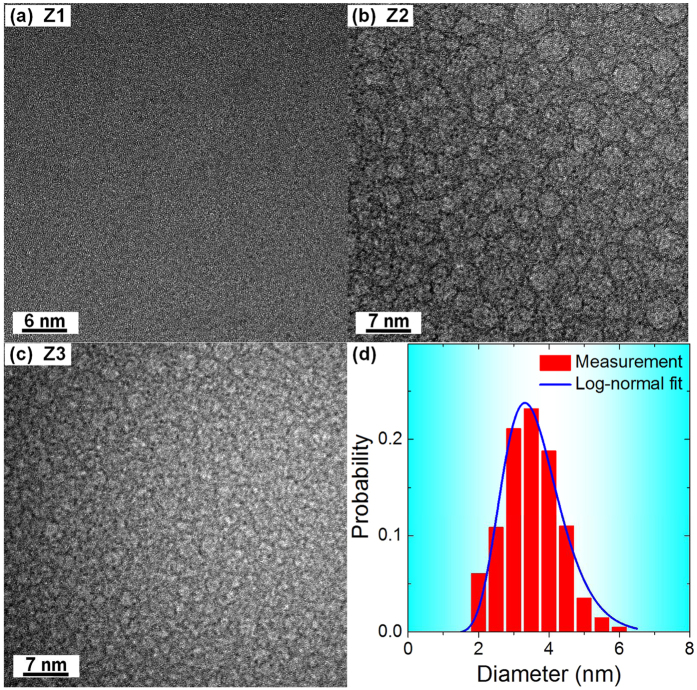
HRTEM micrographs of as-cast specimens. (**a**) Z1 – (Fe_0.6_Cu_0.4_)_33_Al_8_Zr_59_, (**b**) Z2 – (Fe_0.45_Cu_0.55_)_33_Al_8_Zr_59_, and (**c**) Z3 – (Fe_0.3_Cu_0.7_)_33_Al_8_Zr_59_. Panel (**d**) shows the size distribution for the glassy nanospheres in as-cast Z2.

High-resolution TEM (HRTEM) images taken from cross-sectioned as-cast rods with compositions (Fe_0.6_Cu_0.4_)_33_Al_8_Zr_59_, (Fe_0.45_Cu_0.55_)_33_Al_8_Zr_59_, and (Fe_0.3_Cu_0.7_)_33_Al_8_Zr_59_ (hereafter Z1, Z2 and Z3) are presented in Fig. 1. While a uniform contrast is observed in Z1 (Fig. 1(a)), there is a microstructure of nanospheres in Z2 (Fig. 1(b)) and Z3 (Fig. 1(c)). HRTEM and fast Fourier transformation (FFT) patterns (not shown) indicate no evidence for nanocrystals in any of the samples. Glassy structure is further confirmed by X-ray diffraction (XRD) patterns (Fig. S1(a)) as well as by clear glass transitions and crystallization exotherms seen in differential scanning calorimetry (Fig. S1(b)).

For as-cast Z2, the nanosphere diameter is 2–5 nm, approximating to a log-normal distribution (Fig. 1(d)). The population density and volume fraction of the nanospheres are estimated by standard stereological methods to be 5.2 × 10^24^ m^−3^ and 49.3 ± 0.2%. The microstructures of as-cast Z2 and Z3 are consistent with Cu/Fe immiscibility^36^,^37^,^38^ (Fig. S2). The nanoscale of the phase separation in these glasses gives them some similarities with multiphase *nanoglasses* obtained by condensation or sputtering^39^. For as-cast Z3, the glassy nanospheres are so small that their size is difficult to determine (Fig. 1(c)). Comparison of Z2 and Z3, however, suggests that the microstructural scale is tunable by changing the Cu:Fe atomic ratio.

**Figure 2:**
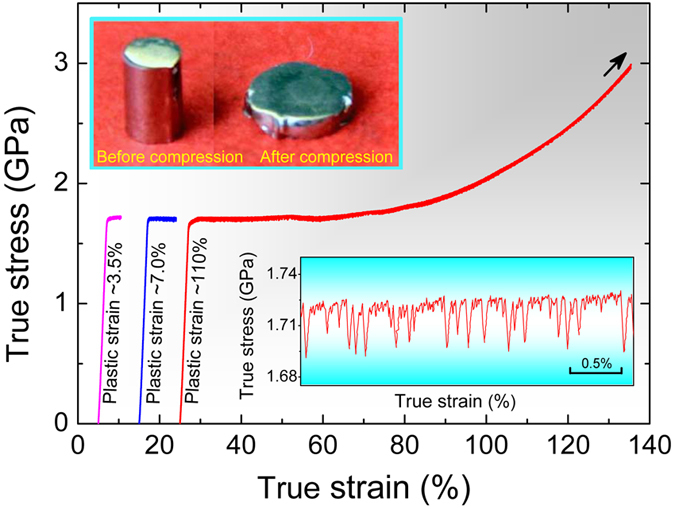
True stress-strain curves of Z2 uniaxially compressed to different plastic strains at room temperature (curves are shifted to the right for clarity). The top-left inset shows a Z2 specimen before and after compression to 110% plastic strain. The bottom-right inset shows a magnified view of the serrated flow in the plastic regime.

Z2 samples (height:diameter = 2:1) were uniaxially compressed to plastic strains of 3.5%, 7.0%, or 110% at room temperature. Figure 2 shows the true stress-strain curves. The rods were compressed to a thin disc without fracture (Fig. 2, inset top-left) when testing was stopped at a plastic strain of 110%. This extraordinary plasticity is comparable with that for bulk MGs and their composites reported elsewhere^27^,^40^,^41^,^42^,^43^,^44^,^45^,^46^,^47^. Such large compressive strains appear to be facilitated by inhomogeneity, for which phase separation, as in the present plastic glasses, is one possible origin.

The shear-band population in Z2 increases with increasing strain (Fig. S3(a,b)), ultimately giving a uniform distribution (Fig. S3(c,d)). Shear-band interactions are also expressed in the complex character of serrations with numerous small stress-drops accompanied by fewer but larger stress-drops (bottom-right inset in Fig. 2)^44^,^45^,^46^. Although phase separation is evident in TEM (Fig. 1), the nanosphere and matrix compositions are rather close, and there is no evidence of two glass transitions, of complex crystallization, or of two diffraction halos (Fig. S1). The nanosphere and matrix phases are very similar, which implies that the present phase-separated structure should deform by the same basic mechanism as a monolithic glass.

**Figure 3:**
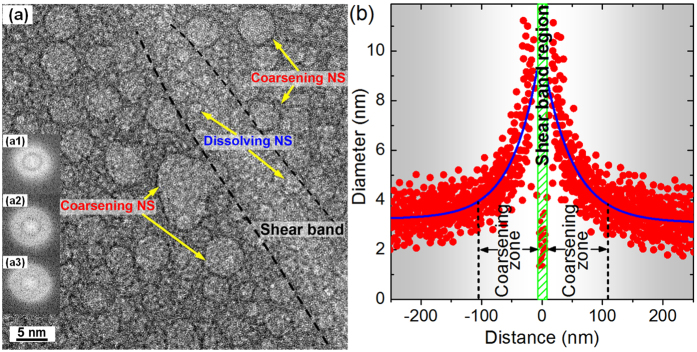
(**a**) HRTEM micrograph taken near a shear band in Z2 deformed to a plastic strain of 7.0%, showing regions of apparent dissolution of the nanospheres (NS) within the band, and coarsening of the nanospheres in the matrix adjacent to the band. Outside the band, the characteristic size of the nanospheres increases with proximity to the shear-band centre, while their population density clearly decreases. Insets (a1), (a2), and (a3) show fast Fourier transformation patterns of the shear-band, nanosphere, and matrix regions, respectively, indicating their glassy nature. (**b**) Typical two-dimensional (2D) statistics of the nanosphere diameter as a function of distance of the individual nanosphere centre from the mid-plane of the shear band. A total of ~600 nanospheres in an area of 500 nm × 250 nm around a typical shear band have been measured.

Figure 3(a) is an HRTEM image of Z2 compressed to 7.0% plastic strain, showing microstructural variation associated with a single shear band (the spacing between bands in this sample is ~50 μm, Fig. S3(b)). There are just a few small nanospheres within the band, and notably larger nanospheres in a zone of about 100 nm on either side (Fig. 3(a,b)). The apparent dissolution of nanospheres inside the band, while preserving the glassy state, contrasts with the reported formation of nanocrystals within shear bands^8^,^12^,^47^,^48^.

**Figure 4:**
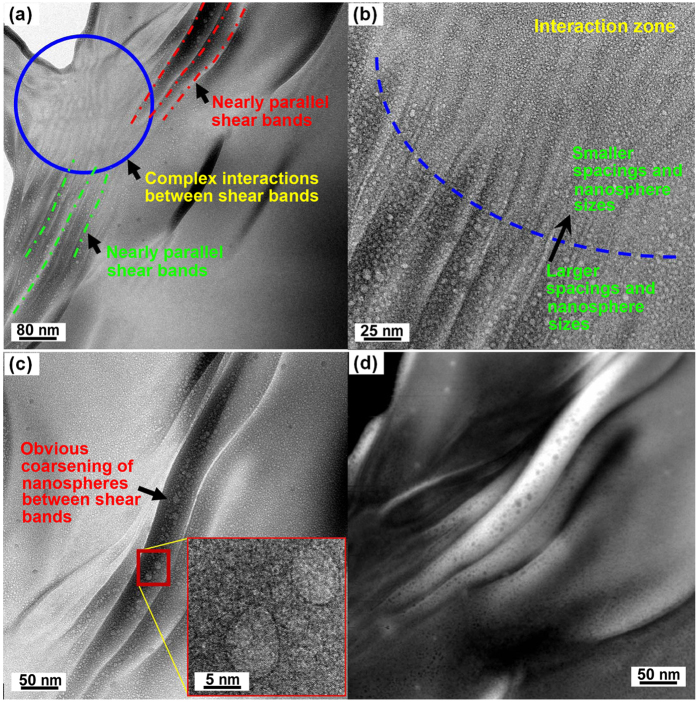
(**a**) TEM image of Z2 sample compressed uniaxially to a thin-disc shape (~110% plastic strain, Fig. 2), showing numerous shear bands (lines) and an interaction zone (circle). (**b**,**c**) HRTEM micrographs of the shear bands near the green and red lines in (**a**). The inset in (**c**) is a higher-magnification image of the region marked by the red rectangle. (**d**) STEM image corresponding to the TEM image in (**c**), further verifying chemical heterogeneity.

Transmission electron microscopy of the Z2 samples compressed to ~110% plastic strain showed a complex pattern of shear bands several micrometres long and with thickness ranging from 3 to 20 nm (Fig. 4). Plastic deformation gives regions with many nearly parallel shear bands. For example, the green and red lines in Fig. 4(a) show shear bands with thickness of several nanometres separated by spacings of 20–50 nm. In regions of more complex interactions (circled), the spacings are estimated to be smaller (15–30 nm, Fig. 4(b)) and the image contrast is then decreased as expected, as there is presumably a smaller shear offset on each band.

Distinct from the general background, in regions close to shear bands, and especially between parallel shear bands, there is obvious coarsening of the glassy nanospheres, as shown within the red rectangle in Fig. 4(c). The scanning TEM (STEM) image (Fig. 4(d)) of deformed Z2 shows that the compositional heterogeneity near shear bands is more pronounced than elsewhere. As determined by energy-dispersive X-ray analysis, the nanospheres are richer in Fe (mean composition Fe_17.4_Cu_14.1_Al_8.3_Zr_60.2_, at.%), whereas the matrix is richer in Cu (mean composition Fe_12.7_Cu_22.5_Al_7.5_Zr_57.3_). In the coarsened microstructure, the nanospheres and the matrix remain fully glassy, as indicated by HRTEM (inset in Fig. 4(c)) and FFT patterns (insets in Fig. 3(a)).

The fully glassy nature of Z2 during and after deformation was confirmed by *in-situ* synchrotron XRD (Fig. S4(a)). The structural change induced by plastic deformation of Z2 was assessed from structure factors *S*(*Q*) and pair-distribution functions *g*(*r*). In *S*(*Q*) the first diffuse maximum is broadened, implying structural disordering (Fig. S4(b)). This is shown more clearly in *g*(*r*), especially in the first coordination shell (Fig. S4(c), including inset), where deformation induces smearing of the peaks at *r* = 2.71 Å and 3.15 Å. Estimation of the mean density from the XRD curves as described elsewhere^49^,^50^ reveals a decrease of ~0.5% upon ~110% plastic strain. This is similar to the ~0.6% density decrease seen in a MG subjected to large strain in high-pressure torsion^23^.

## Discussion

The cusp-shape profile of the nanosphere diameter (Fig. 3(b)) is reminiscent of the microhardness profile^10^ with a softened plane up to ~160 μm thick across a single shear band. The thickness of the softened zone was proportional to the imposed strain, being roughly the same as the shear offset on the band. If the shear-band offset is similar to the width of the profile in Fig. 3(b), then the offset, estimated as ~100 nm, is so short that it would be firmly in the range where there is no significant heating^51^.

In contrast to measured hardness profiles^10^, the present HRTEM permits identification of a microstructurally distinct central zone (~15 nm thick in Fig. 3(b)). Given that MGs show a decrease of viscosity under shear, flow should be concentrated in a very thin band, consistent with this central zone. In this zone, it is unlikely that the nanospheres have been destroyed by heating into the single-phase field of the liquid followed by rapid quenching. Rather, the probable mechanism is direct intense mechanical shearing and mixing in the solid, processes associated with amorphization rather than crystallization.

**Figure 5:**
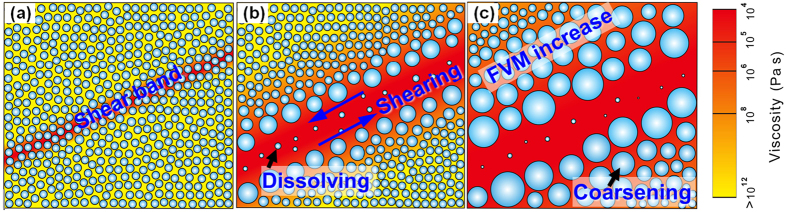
Schematic of the deformation-induced structural evolution in the phase-separated bulk MGs. (**a**) Onset of a shear band in the first stages of loading. (**b**) Dissolving of glassy nanospheres inside the shear band due to mechanical shearing and mixing. (**c**) The increased free volume and mobility (FVM) in the matrix near a shear band are generated by relatively homogeneous flow in the matrix itself. Coarsening of the nanospheres near the shear band is by Ostwald ripening enabled by a local increase in mobility.

The coarsened microstructure near the shear band (Fig. 3) does not show any evidence for shear-induced nanoparticle movement and coalescence as found in a deformed MG with nanocrystals^52^ or in low-viscosity systems^53^. Though some coalescence cannot be excluded, the coarsening is likely to be mainly by Ostwald ripening of the nanospheres enabled by a local increase in mobility, which we now attempt to quantify. We assume that coarsening occurs throughout the 280 s loading time corresponding to the imposed 7.0% plastic strain for the sample in Fig. 3. Nanospheres of average 3.3 nm diameter coarsen to ~10 nm adjacent to the shear band (Fig. 3b). A standard analysis of ripening, with parameters estimated as in Supplementary Information, leads to a rough estimate that the solute diffusivity necessary to give the observed coarsening is ~3 × 10^−18^ m^2^ s^−1^. This can only be regarded as a rough estimate, within 1 to 2 orders of magnitude of the actual value. Interestingly, the value is close to the *D* = (1 to 2) × 10^−17^ m^2^s^−1^ found for tracer diffusion in shear bands; this was estimated to be some 8 orders faster than in the undeformed matrix^15^. From the Stokes-Einstein relation^15^,^54^ this diffusivity would correspond to a viscosity of ~6 × 10^5^ Pa s (Supplementary Information). That this inferred viscosity, immediately adjacent to the shear band, is within the range <10^4^ to 10^8^ Pa s already identified for shear-band operation^55^ suggests that the assumption of Ostwald ripening is reasonable. The deformation-induced structural evolution in the phase-separated bulk MGs is sketched in Fig. 5.

The increased free volume and mobility in the matrix near a shear band may be generated by relatively homogeneous flow in the matrix itself. Though the operating time of individual shear bands can be short, flow in the intervening matrix, necessary to ensure strain compatibility in the material as a whole, is likely to be more spread over time; thus free volume may be generated throughout the full deformation time. It is known from work on elastostatic loading of bulk MGs that free volume can be generated very efficiently by homogeneous plastic flow at room temperature. For example, a bulk MG subjected to a compressive homogeneous plastic strain of only 2.3 × 10^–5^ at room temperature showed a density decrease of 0.26%^56^, roughly half of the overall density decrease seen after 110% plastic strain in the present work. Although the strain in shear bands is very much higher, its effects saturate after small strain and it does not lead to proportionally large property changes.

It is not reasonable to assume that a shear band spanning a substantial sample cross-section would be atomically flat. When it operates, then, there must be some smoothing out of the topography of the shear-band plane. The plastic deformation necessary to achieve this would obviously be greatest adjacent to the shear band itself. In this way we can understand how the cusp-shaped profiles can arise, and how the profiles must thicken as shear continues to larger offsets.

As an alternative, a question whether the cusp-shaped profiles could arise from free volume diffusing out from the central zone of the shear band is discussed here. For the profile in Fig. 3(b), taking the diffusion time (as above) of 280 s, the diffusivity to achieve a profile half-width of ~100 nm is ~10^−17^ m^2^s^−1^, higher than the value that would be compatible with the observed ripening. For the thicker profiles^10^, it would require values of diffusivity that seem unphysically high. From this viewpoint, the local generation of free volume within the matrix is more likely than diffusion outwards from the shearing zone^57^,^58^,^59^.

In summary, we have synthesized new bulk phase-separated MGs containing a high population density of nanospheres of a diameter (2–5 nm) fine enough to act as a local internal recorder of plastic flow. The glassy nanospheres inside the shear band dissolve through mechanical mixing driven by the sharp strain localization there, while those nearby in the matrix coarsen by Ostwald ripening due to the increased atomic mobility. Our work presents direct visual evidence for deformation-related effects, in particular increased atomic mobility, in the region around shear bands. These findings demonstrate that there exists a thicker affected zone around the shear band, indicating that the measured property changes derive from not only the shear bands but also from the adjacent matrix.

## Methods

### Sample preparation

[Fe_*x*_Cu_(1–*x*)_]_33_Al_8_Zr_59_ (*x* = 0.3, 0.45, 0.6) alloys (at.%) were prepared by arc-melting metals with high purities (at least 99.8%) under a Ti-gettered argon atmosphere in a water-cooled copper crucible. The alloys were remelted several times, and rods (1.5 mm diameter, 50 mm length) were suction-cast in a unit attached to the arc-melter. TEM specimens were ion-milled using a Gatan 691 Precision Ion Polishing System with liquid-nitrogen cooling.

### Mechanical testing

Uniaxial compression tests were performed using an Instron 5869 machine at a strain rate of 2.5 × 10^−4^ s^−1^. Cylindrical samples (1.5 mm diameter) were cut to 3 mm length, and the rod-ends were ground, polished and lubricated. The strain *ε* was measured directly on the sample using a laser extensometer (Fiedler Optoelektronik). The true strain was calculated as *ε*_true_ = ln(1 + *ε*) and the true stress as *σ*_true_ = *σ*(1 + *ε*) where *σ* = *F*/*A*_i_ (*F* – applied load and *A*_i_ – initial area of the cross-section).

### Structural characterization

The samples were first examined by X-ray diffraction (PANalytical X’Pert Pro instrument, reflection geometry, monochromatic Co-radiation). Chemical heterogeneity was investigated by STEM with a resolution of 0.2 nm and energy-dispersive analysis (EDX), using a high-resolution instrument (HRTEM: Philips Tecnai G2 F20, accelerating voltage 200 kV). The thermal behaviour (glass transition, crystallization) was studied using a differential scanning calorimeter (Perkin Elmer DSC 7) at a heating rate of 20 K·min^–1^ under a continuous flow of argon.

### High-energy synchrotron XRD

The structures of as-cast and plastically deformed samples of the Z2–(Fe_0.45_Cu_0.55_)_33_Al_8_Zr_59_ and Z3–(Fe_0.3_Cu_0.7_)_33_Al_8_Zr_59_ glasses were studied using high-energy synchrotron XRD at the P07 beamline at the PETRA III storage ring (DESY, Hamburg, Germany). *In-situ* XRD under uniaxial compression was carried out using a Kammrath & Weiss test rig at a strain rate of 1.0 × 10^−4^ s^−1^. The measurements were performed in transmission with an incident-beam photon energy of 100 keV and a beam size of 0.25·0.25 mm^2^. Two-dimensional XRD patterns recorded by a Perkin Elmer 1621 detector were processed using the FIT2D program. A LaB_6_ standard placed at the sample position was measured as a calibrant. After corrections for background, polarization, absorption, fluorescence, and incoherent scattering, the XRD intensities were converted to Faber-Ziman structure factors *S*(*Q*) and pair-distribution functions *g*(*r*).

## Additional Information

**How to cite this article**: He, J. *et al.* Local microstructure evolution at shear bands in metallic glasses with nanoscale phase separation. *Sci. Rep.* **6**, 25832; doi: 10.1038/srep25832 (2016).

## Supplementary Material

Supplementary Information
