# Supplementary material for: Local microstructure evolution at shear bands in metallic glasses with nanoscale phase separation
Source: Sci Rep. 2016 May 16;6:25832. doi: 10.1038/srep25832 (PMC4867588; doi:10.1038/srep25832)
Supplement: Supplementary Information [file srep25832-s1.pdf]

Supplementary Information

Local microstructure evolution at shear bands in metallic glasses with nanoscale phase separation

Jie He[1,2], Ivan Kaban[2,3], Norbert Mattern[2], Kaikai Song[2], Baoan Sun[2], Jiuzhou Zhao[1], Do Hyang Kim[4], Jürgen Eckert[5,6] & A. Lindsay Greer[7,8]

[1]Institute of Metal Research, Chinese Academy of Sciences, Shenyang 110016, China.

[2]IFW Dresden, Institute for Complex Materials, PO Box 270116, Dresden 01171, Germany.

[3]TU Dresden, Institute of Materials Science, Dresden 01062, Germany.

[4]Department of Metallurgical Engineering, Center for Noncrystalline Materials, Yonsei University, Seoul 120-749, Korea.

[5]Erich Schmid Institute of Materials Science, Austrian Academy of Sciences, Jahnstraβe 12, A-8700 Leoben, Austria.

[6]Department Materials Physics, Montanuniversität Leoben, Jahnstraβe 12, A-8700 Leoben, Austria.

[7]Department of Materials Science & Metallurgy, University of Cambridge, Cambridge CB3 0FS, UK.

[8]WPI Advanced Institute for Materials Research (WPI-AIMR), Tohoku University, Sendai 980-8577, Japan.

Correspondence and requests for materials should be addressed to J.H. (email: jiehe@imr.ac.cn), or I.K. (email: i.kaban@ifw-dresden.de), or A.L.G. (email: alg13@cam.ac.uk)

Supplementary Figures

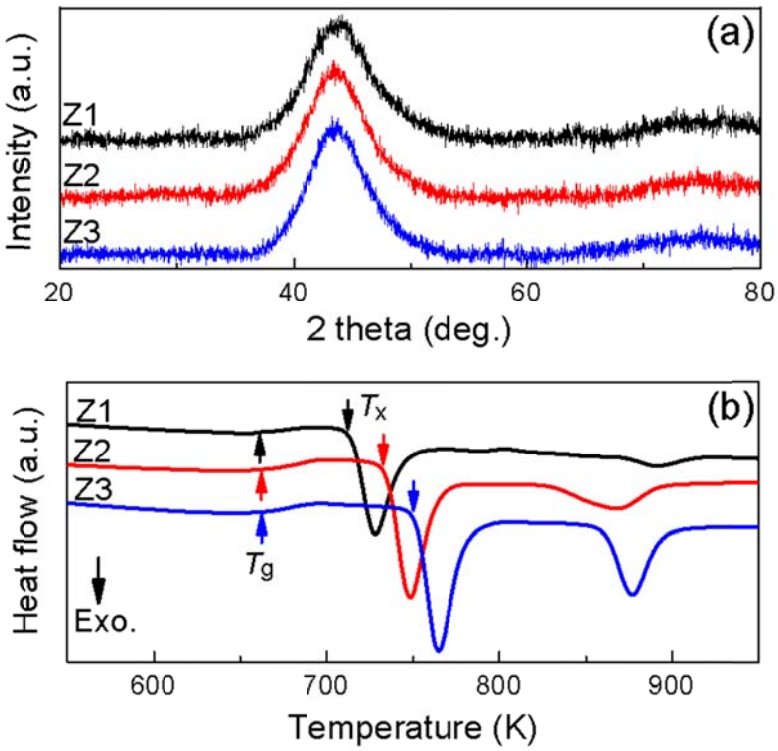

Figure S1. (a) XRD patterns of the rods with compositions Z1–$(Fe_{0.6}Cu_{0.4})_{33}Al_8Zr_{59}$, Z2–$(Fe_{0.45}Cu_{0.55})_{33}Al_8Zr_{59}$, and Z3–$(Fe_{0.3}Cu_{0.7})_{33}Al_8Zr_{59}$, show broad halos and no Bragg peaks; (b) differential scanning calorimetry traces for samples heated at 20 $K{\cdot}min^{-1}$, show the glass transition at T_g and crystallization exotherms with onset at T_x. In each case, the glassy nature of the as-cast samples is confirmed.

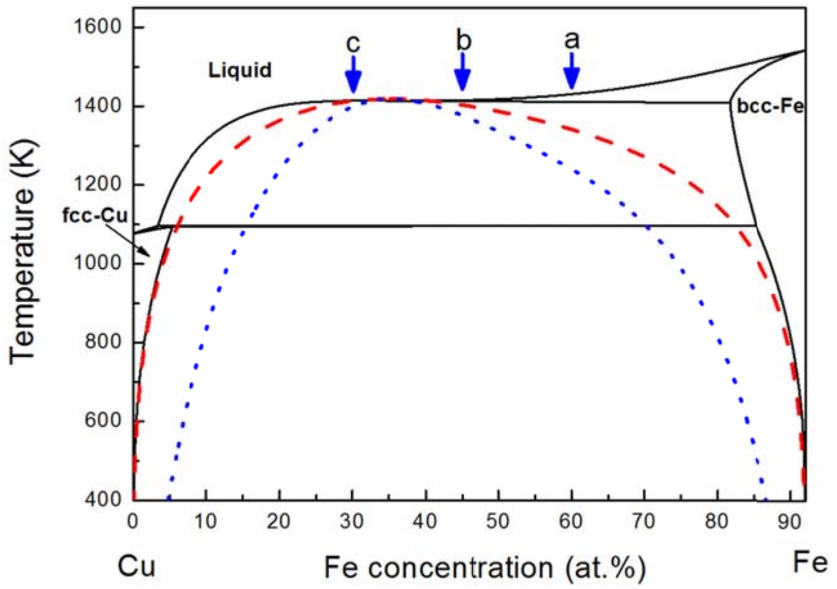

Figure S2. Phase diagram for the $[Fe_xCu_{(1-x)}]_{92}Al_8$ cross-section, calculated using thermodynamic data of Miettinen[S1]. The *dashed red* and *dotted blue* curves are bimodal and spinodal lines, respectively. The *blue* arrows point to the compositions of the cast $[Fe_xCu_{(1-x)}]_{92}Al_8$ rods with (a) x = 0.6; (b) x = 0.45; (c) x = 0.3.. The critical point composition is $[(Fe_{0.35}Cu_{0.65})_{92}Al_8]$.

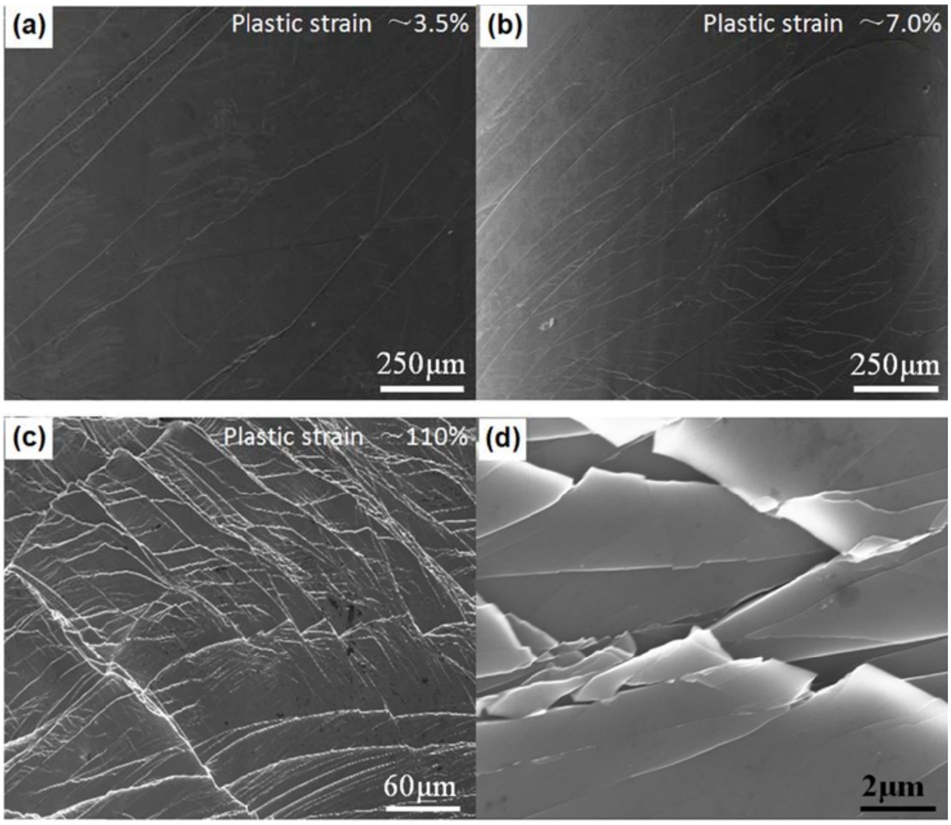

Figure S3. SEM side-view image of Z2–$(Fe_{0.45}Cu_{0.55})_{33}Al_8Zr_{59}$ specimens, compressed to (a) ~3.5%, (b) ~7.0%, and (c) ~110% plastic strain; (d) locally magnified image of panel (c). The wavy and differently oriented shear bands indicate the complexity of maintaining strain continuity in the sample.

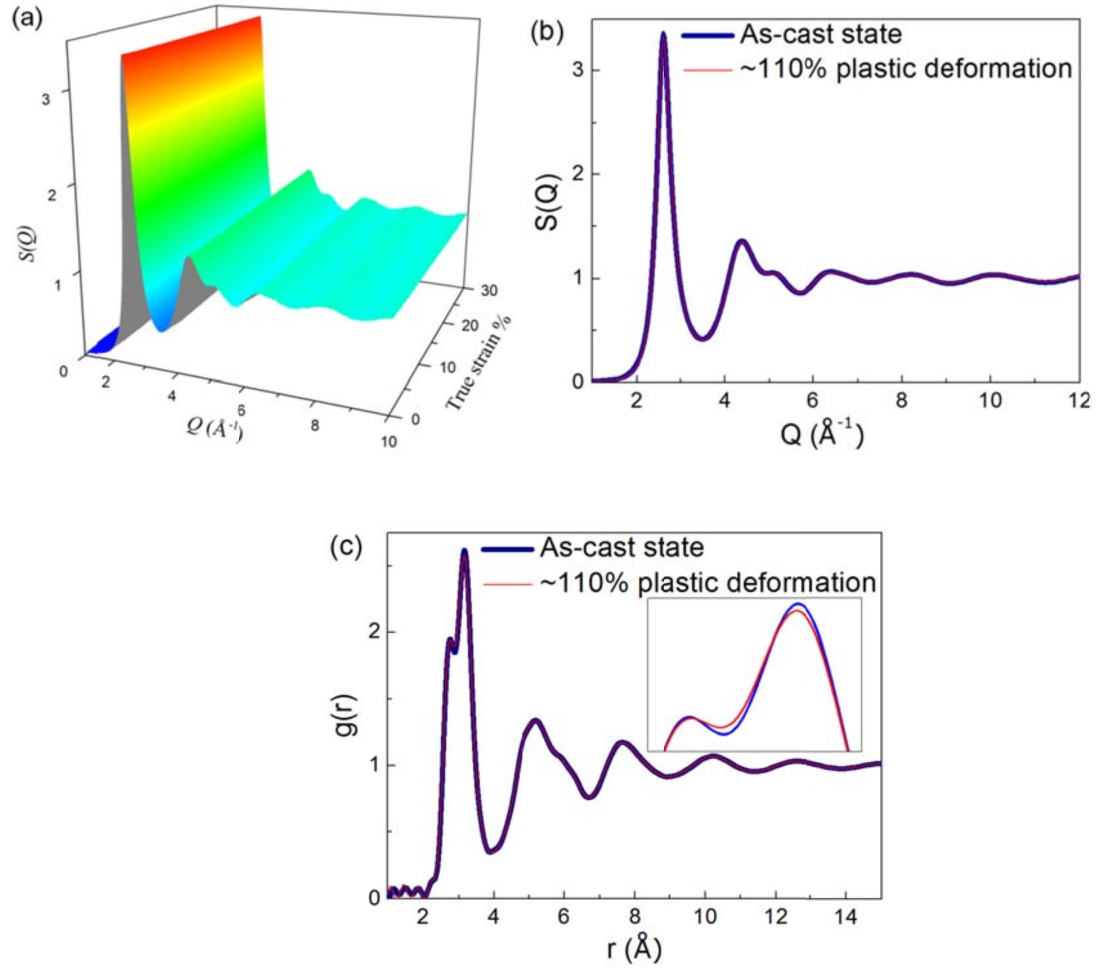

Figure S4. Synchrotron X-ray diffraction. (a) Structure factors for a Z2 – $(Fe_{0.45}Cu_{0.55})_{33}Al_8Zr_{59}$ sample, measured in-situ by high-energy synchrotron diffraction during uniaxial compression; (b) structure factors, and (c) total pair-distribution functions for 0 and 110% plastic strain. The *thick blue* and *thin red* lines show measurements on specimens, respectively, in the as-cast state and after ~110% plastic deformation. The inset in (c) is a magnified image of the first peak. The peaks at *r* = 2.71 Å and *r* = 3.15 Å, correspond, respectively, to the nearest-neighbor bonds of the smaller Cu and Fe atoms (with all of their Cu, Fe and Zr neighbors) and the longer Zr-Zr bonds[S2]. In addition to the smearing of these peaks, the Zr-Zr peak slightly decreases and shifts to smaller *r*-values.

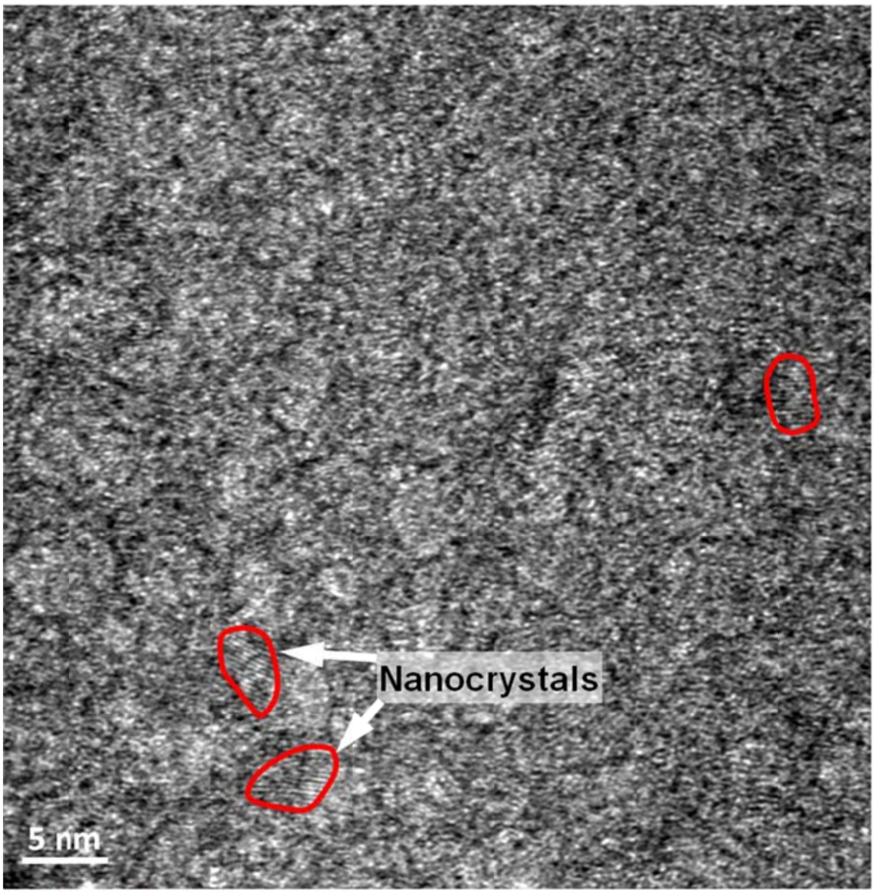

Figure S5. HRTEM micrograph of annealed Z2 sample, indicating that there may be some crystallization as marked.

Supplementary Note

The observed coarsening of the nanosphere dispersion near a shear band is analyzed as Ostwald ripening, with the intention that the kinetic analysis can yield a value for the effective solute diffusion coefficient *D* in the metallic-glass matrix. The kinetics of Ostwald ripening in the case where the dispersed phase occupies a significant volume fraction has been treated by Marqusee and Ross[S3]. In the limiting case of zero volume fraction of dispersed phase, the average particle radius $\bar{r}$ of the dispersed phase increases with time *t* according to:

$$\bar{r} = \left(\frac{8\gamma V_{\mathrm{m}} X_{\mathrm{eq}}^{\infty}}{9RT} \right)^{1/3} D^{1/3} t^{1/3} \qquad \text{(S1)}$$

where γ is the energy per unit area of the interface between the dispersed spheres and the matrix, V_{m} is the molar volume of the dispersed phase, X_{eq}^{∞} is the mole fraction of solute that would be in equilibrium in the matrix at a planar interface with the dispersed phase, *R* is the gas constant and *T* is the temperature. Marqusee and Ross show that coarsening is accelerated for finite volume fractions[S3], but the effect is well within one order of magnitude and we ignore it in the present work. The parameters in Equation S1 are largely unknown for the metallic-glass systems in the present work, and are estimated as follows.

Phase separation has been studied for the Cu-Fe system[S4], where γ was taken to be $0.16\ \mathrm{J\ m^{-2}}$ for spheres in a liquid matrix and $0.43\ \mathrm{J\ m^{-2}}$ for spheres in a solid matrix. In the present case of a metallic glass, we take $\gamma = 0.3\ \mathrm{J\ m^{-2}}$. From the range of values for similar compositions[S5], we take $V_{\mathrm{m}} = 11.4 \times 10^{-6}\ \mathrm{m^3\ mol^{-1}}$. As noted in the main text, Cu and Fe are the elements principally involved in the phase separation and Fe is concentrated in the

nanospheres. We take Fe to be the solute for the kinetic analysis and take its measured level in the matrix, ~13%, as an estimate of X_{eq}^{∞}.

We assume that the coarsening of nanospheres takes place at room temperature (298 K) throughout the time of plastic deformation, which is 280 s for 7.0% strain at the imposed strain rate of of $2.5\times10^{-4}\ \mathrm{s}^{-1}$. The profile in Figure 3b shows that nanospheres with an average diameter of 3.3 nm coarsen to an average of ~10 nm adjacent to the shear band. Using the corresponding values of $\bar{r}$ in Equation S1, we solve for D and obtain a value of ~ $3\times10^{-18}\ \mathrm{m}^2\ \mathrm{s}^{-1}$.

To estimate the associated viscosity η, we use the Stokes-Einstein relation[S6]:

$$\eta = \frac{kT}{3\pi a D} \quad \text{(S2)}$$

where a is an effective atomic diameter or jump distance, and k is Boltzmann's constant. From the Goldschmidt atomic radii for Cu and Fe, we take $a = 0.256\ \mathrm{nm}$. Then the estimated viscosity for the coarsening regime is $\sim 6\times10^{5}\ \mathrm{Pa\ s}$. The Stokes-Einstein relation is valid for high temperature liquid melt, and there is evidence showing it is still applicable for the relatively low temperature glassy solid[S7, S8].

Increased mobility might arise from a local increase in temperature rise. The Z2 sample was annealed at 573 K (i.e. at 86% of T_g) for 30 minutes; this did not result in any detectable coarsening of the nanospheres, but there may be some crystallization (Figure S5). As noted in the main text, it is not considered likely in any case that there is any significant temperature increase. Rather, the increased mobility is interpreted as an effect of homogeneous plastic

deformation at room temperature. It is known that such deformation of metallic glasses can have a significant rejuvenation effect, increasing free volume and mobility.

S1. Miettinen J. Thermodynamic description of the Cu-Al-Fe system at the Cu-Fe side. Calphad **27,** 91-102 (2003).

S2. Kaban, I. *et al*. On the atomic structure of $Zr_{60}Cu_{20}Fe_{20}$. *J. Phys. Cond. Matter* **22,** 404208 (2010).

S3. Marqusee, J. A. & Ross, J. Theory of Ostwald ripening: competitive growth and its dependence on volume fraction. *J. Chem. Phys.* **80,** 536–543 (1984).

S4. He, J., Zhao, J. Z. & Ratke, L. Solidification microstructure and dynamics of metastable phase transformation in undercooled liquid Cu-Fe alloys. *Acta Mater.* **54,** 1749–1757 (2006).

S5. Wang, W. H. The elastic properties, elastic models and elastic perspectives of metallic glasses. *Prog. Mater. Sci.* **57,** 487–656 (2012).

S6. Battezzati, L. & Greer, A. L. The viscosity of liquid-metals and alloys. *Acta Metall.* **37,** 1791–1802 (1989).

S7. Bokeloh, J., Divinski, S. V., Reglitz, G. & Wilde, G. Tracer measurement of atomic diffusion inside shear bands of a bulk metallic glass. *Phys. Rev. Lett.* **107,** 235503 (2011).

S8. Bartsch, A., Rätzke, K., Meyer, A. & Faupel, F. Dynamic arrest in multicomponent glass-forming alloys. *Phys. Rev. Lett.* **104,** 195901 (2010).
